# The effect of a static magnetic field and baicalin or baicalein interactions on amelanotic melanoma cell cultures (C32)

**DOI:** 10.1007/s11033-022-07148-z

**Published:** 2022-01-20

**Authors:** Agnieszka Synowiec-Wojtarowicz, Katarzyna Pawłowska-Góral, Agata Krawczyk, Stanisław Gawron, Magdalena Kimsa-Dudek

**Affiliations:** 1 Department of Nutrigenomics and Bromatology, Faculty of Pharmaceutical Sciences in Sosnowiec, Medical University of Silesia, Katowice, 8 Jednosci Street, 41-200 Sosnowiec, Poland; 2 Institute of Electrical Drives and Machines KOMEL, 188 Rozdzienskiego Street, 40-203 Katowice, Poland

**Keywords:** Baicalin, Baicalein, Static magnetic fields, Interactions, Redox homeostasis, Melanoma cells

## Abstract

**Background:**

Baicalin and baicalein have antioxidant, anti-inflammatory, hepatoprotective and anti-cancer properties. However, it is not known how a static magnetic field will modify these properties. Therefore, the aim of our study was to evaluate the simultaneous exposure of melanoma cells to flavones and the static magnetic fields that are generated by permanent magnets on the gene expression and the activity of the antioxidant enzymes that are associated with the antioxidant defense system.

**Methods and results:**

Melanoma cells that had been treated with baicalin or baicalein were subjected to a static magnetic fields with a moderate induction. The static magnetic field was emitted by permanent magnets and the cell cultures were carried out in special test chambers. The research included determining the activity of the antioxidant enzymes (superoxide dismutase, glutathione peroxidase and catalase) as well as the gene expression profile. The addition of the flavones to the cell cultures at a concentration of 50 µmol/L resulted increase in the expression of the *SOD1*, *SOD2* and *GPX1* genes compared to the nontreated cell cultures. Simultaneous exposure of the melanoma cells to static magnetic field and baicalin or baicalein reduced their mRNA levels compared to the cultures to which only baicalin or baicalein had been added. The change in gene expression was accompanied by changes at the protein level associated with an increase in the activity of antioxidant enzymes.

**Conclusion:**

We showed that baicalin or baicalein have anticancer properties by disturbing the redox homeostasis in melanoma cells and also increases the antioxidant system gene expression. There was also an antagonistic interaction between the studied flavones and the static magnetic field, which cause a decrease in the anticancer effects of baicalin or baicalein.

## Introduction

The growing human exposure to factors that induce oxidative stress has resulted in an increase in consumer curiosity about food that contains antioxidant substances. These compounds support the body's natural defenses in the fight against free radicals, an excess of which promotes the development of many diseases such as atherosclerosis, arterial hypertension, neurodegenerative diseases or cancer [1]. Vegetables, fruits and coffee and tea, which are commonly consumed, are excellent sources of antioxidant substances. Baicalin and baicalein, which belong to the flavones, constitute a characteristic group of compounds that present in various species of *Scutellaria* such as *Scutellaria baicalensis* and *Scutellaria galericulata* [2].

The research confirmed that these compounds exhibit a multidirectional effect on the molecular processes: they inhibit the development of neoplasms, have neuroprotective and hepatoprotective properties as well as antioxidant and anti-inflammatory properties. The antioxidant properties of poliphenols depend on the number of hydroxyl groups in the molecule, the more the stronger these properties. Due to the presence of hydroxyl gropus, baicalein and baicalin exhibit strong antioxidant properties that consists in a direct reaction with free radicals or the chelation of transition metal ions, which protects the body against the formation of a very reactive hydroxyl radical [3]. Baicalin and baicalein also have a potent anticancer activity, which is not only cytostatic but also cytotoxic to various human tumor cell lines in vitro. The antitumor functions of these flavones are largely due to their ability to scavenge oxidative radicals, to attenuate the NF-κB activity, to inhibit several genes that are important for regulating the cell cycle and to suppress *COX-2* gene expression [4].

The anti-inflammatory and immunostimulatory effect of these flavonoids results from their ability to inhibit lymphocyte proliferation, inhibit the synthesis of IgE, IgM and IgG antibodies and release cytokines. Moreover, baicalein can inhibit the activity of the lysosomal enzymes, which are involved in the inflammatory and allergic processes and additionally inhibits the secretion of eotaxin, which is secreted by fibroblasts [5]. Studies have also confirmed the antifungal activity of flavones against many human pathogens and their antibacterial activity has been demonstrated against the bacteria that cause an inflammation of the oral cavity [6].

Magnetic fields are common in the natural environment and the effects of their influence on the human body have been the subject of many studies. The presence of magnetic fields in everyday life carries the risk of functional disorders in the cells and tissues and biological systems [7]. The research results have not yet provided a clear answer about the positive or negative effects of a static magnetic field on the human body. Magnetic fields have been successfully used in medicine for many years in diagnosing and treating diseases of the musculoskeletal system, nervous system, organs of vision, the gastrointestinal tract, skin and soft tissues. The basis for the use of a magnetic field in medicine is its participation in the regeneration processes of soft tissues, as well as its anti-inflammatory and anti-swelling properties [8]. However, there are many studies that have determined that a magnetic field may contribute to the generation of free radicals and, as a result, induce oxidative stress, which results in a disturbance of the cellular redox homeostasis [9].

Due to the growing use of biologically active substances, which have been known as components of drugs, dietary supplements or cosmetic preparations for many years and the search for new ways of using them in chemotherapy, the main aim of our study was to assess the impact of the simultaneous exposure of melanoma malignant cells to baicalin or baicalein and the static magnetic fields that are generated by permanent magnets on the gene expression profile related to the antioxidant defense system and on the activity of the antioxidant enzymes. It is not yet known whether a static magnetic field will modify the action of baicalin and baicalein on melanoma cells. One of the most malignant skin cancers is melanoma it has a high metastatic potential as well as a high resistance to treatment, which results in a high mortality rate. The basic method for treating early-stage melanomas is surgery alone. If surgery is not possible or as an adjuvant therapy, radiotherapy, chemotherapy is rarely used because of its low response rate. Therefore, it seems advisable to consider physical options such as using a moderate-intensity static magnetic field (SMF) or the bioactive compounds that are found in foods as potential agents that can be used to support cancer therapy.

## Material and methods

### Reagent and chemicals

DMEM (Dulbecco's Modified Eagle Medium)—Lonza, Fetal Bovine Serum—Lonza, Penicillin—Sigma Aldrich, Amphotericin B—Sigma Aldrich, Trypsin EDTA solution—Lonza, 0.4% Trypan Blue—Invitrogen, DMSO (dimethyl sulfoxide)—Sigma Aldrich, PBS (buffered saline solution)—Lonza, Baicalin—Sigma Aldrich, Baicalein—Sigma Aldrich, Protease inhibitor—Sigma Aldrich, Phosphatase inhibitor—Sigma Aldrich, TRIzol reagent—Invitrogen.

### Equipment

Incubator—Haraeus, Countess TM Automated Cell Counter—Invitrogen, Centrifuge—MPW 223e, Incubator—Incucell, Nano MN-913″—Maestrogen, DNA Engine Opticon™ System—MJ Research Inc, Multi-functional microplate reader—Victor.

### Cell culture conditions

Amelanotic melanoma cells (C32 cell line) were obtained from ATCC (CRL-1585; Manassas, VA, USA) and routinely maintained in a DMEM medium, which was supplemented with 10% fetal bovine serum, penicillin (10,000 U/ml) and amphotericin B (0.25 mg/mL) at 37 °C in a 5% CO_2_ incubator (Heraeus).

Both the number of cells and their viability were monitored by cell counting in a Countess TM Automated Cell Counter (Invitrogen, USA) after staining with 0.4% trypan blue. The experiment was performed on cells that were in the logarithmic phase of growth under conditions of ≥ 98% viability as assessed by the trypan blue exclusion. For the experiments, the melanoma cells were used at three to five passages. The control melanoma cells and melanoma cells that had been treated with baicalin or baicalein were then subjected to a static magnetic field. The baicalin and baicalein concentration was selected with 0.1–250 µmol/L in a pilot study. The concentration 50 µmol/L was selected for the experiment because it was cytotoxic for the melanoma cells and non-cytotoxic for the skin fibroblasts (NHDF cell line). The addition of 50 µmol/L baicalin or baicalein to melanoma cells culture reduced cell viability by about 20% compared to the control [10]. Melanoma cells were cultured in culture flasks. One million cells were introduced into each flask, and after 24 h incubation, they were treated with 50 µmol/L baicalin or baicalein and placed in test chambers emitting a static magnetic field with an induction of 0.7 T. The cultures were maintained in the test chambers at 37 °C in a 5% CO^2^ incubator (Heraeus) for 24 h. Next, the cells were washed with PBS and the cell numbers were determined by cell counting in a Countess TM Automated Cell Counter (Invitrogen, USA) after staining with 0.4% trypan blue.

### Exposure of melanoma cells to static magnetic fields

To study the cells in a static magnetic field, magnetic chambers composed of permanent magnets and a ferromagnetic yoke were used (patent P-396639). The ferromagnetic yoke is the bottom and cover of the chamber and there is a window in the front wall of the chamber that is matched to the dimensions of the cell culture flask [11]. A homogeneous distribution of the magnetic induction over the surface of the flask is conditioned by the structure of the test chamber. The static magnetic field is generated by neodymium magnets and the magnetic field intensity is proportional to the magnetic field strength. The chambers are constructed with the following materials: N42SH magnets, Br = 1.28–1.34 T, HcB ≥ 955 kA/m, HcJ ≥ 1512 kA/m, (BH)max = 310–342 kJ/m^3^, S235JR steel and a diamagnetic material. The maximum operating temperature of the chambers is 150 °C. A chamber with a field induction of 0.7 T was used for the tests, which was checked with a gauss meter before each experiment. The control chamber is made of steel instead of permanent magnets and the field induction in this chamber is 0 T.

### Preparing the cell lysates

After 24 h, the cells were detached from the surface of the culture vessel using a trypsin/EDTA solution. After trypsin neutralization, the cells were centrifuged for 10 min at 2000 RPM, the supernatant was removed, and the cell pellets were washed with a PBS solution and used to prepare the cell lysates. The composition of the lysis buffer was a protease inhibitor (1.4 mg) and a phosphatase inhibitor (10 µL) that had been dissolved in 1 mL of PBS. The tubes containing the suspension of studied material in a lysis buffer were placed in liquid nitrogen for 30 min and stored at − 80 °C until further analysis. All of the studied biochemical parameters were recalculated for 10^6^ cells.

### Determining the superoxide dismutase activity (SOD)

SOD activity was determined using a Cayman Chemical's Superoxide Dismutase Assay Kit (Cayman Chemical, USA) and were performed according to the manufacturer's protocol. Previously prepared cell lysates were used as the test material, centrifuged after thawing and the collected supernatant was used for further studies.

The SOD activity was determined spectrophotometrically at 440–460 nm. Xanthine and hypoxanthine generate superoxide radicals which, when bound with a tetrazolium salt, transform it into red formazan. One unit of SOD activity is defined as the amount of the enzyme that is needed to convert 50% of the superoxide radicals.

### Determining the glutathione peroxidase activity (GPx)

GPx activity was determined using a Cayman Chemical's Glutathione Peroxidase Assay Kit (Cayman Chemical, USA) and were performed according to the manufacturer's protocol.

The GPx activity was also evaluated based on the spectrophotometric method. The GPx activity was indirectly measured using a coupled reaction with glutathione reductase (GR (glutathione reductase). GPx catalyzes the reduction reaction of cumene hydroperoxide and as a result, an oxidized form of glutathione is formed, which was then reduced in the presence of GR and NADPH oxidation to NADP + (accompanied by a decrease in absorbance). The decrease in A340 absorbance is directly proportional to the GPx activity in the sample.

### Determining the catalase activity (CAT)

The CAT activity was measured using a Catalase Assay Kit (Cayman Chemical, USA). The method is based on the reaction of CAT with methanol in the presence of an optimal concentration of H_2_O_2_. The formaldehyde that was produced was measured spectrophotometrically using 4-amino-3-hydrazino-5-mercapto-1,2,4-triazole as the chromogen. Purpald specifically forms a bicyclic heterocycle with aldehydes, which upon oxidation changes from colorless to purple.

### RNA extraction

Total RNA was extracted using a TRIzol reagent. The RNA extracts were treated with DNase I (RNeasy Mini Kit, Qiagen, Valencia, CA, USA) according to the manufacturer’s instructions. The quality of the extracts and the RNA concentration were determined as was previously reported [12].

### Quantitative real-time RT-PCR assay

The gene expression of *SOD1 (superoxide dismutase 1)*, *SOD2 (superoxide dismutase 2)*, *GPX1 (glutathione peroxidase 1)*, *GSR (glutathione reductase)*, *CAT (catalase)* and *β-actin* were evaluated using real-time RT-qPCR and SYBR Green I chemistry (SYBR Green Quantitect RT-PCR Kit; QIAGEN, Valencia, CA, USA) as was previously described [13].

All of the samples were tested in triplicate. *β-actin* was also included as an endogenous positive control of the amplification and integrity of the extracts in order to monitor the RT-qPCR efficiency. Wells that did not contain a template were run as the negative controls. The thermal profile for the one-step RT-PCR was as follows: reverse transcription at 50 °C for 30 min, denaturation at 95 °C for 15 min and 40 cycles consisting of the following temperatures and time intervals: 94 °C for 15 s, 60 °C for 30 s and 72 °C for 30 s.

### Statistical analyses

All data are expressed as the mean ± the standard deviation. An ANOVA and Tukey’s post hoc test were used to evaluate the results of the experiments. The statistical calculations were performed using STATISTICA 12.0 and the statistical significance was defined at *p* < 0.05.

## Results

### Effect of baicalin or baicalein and SMF on the antioxidant gene expression

**Fig. 1:**
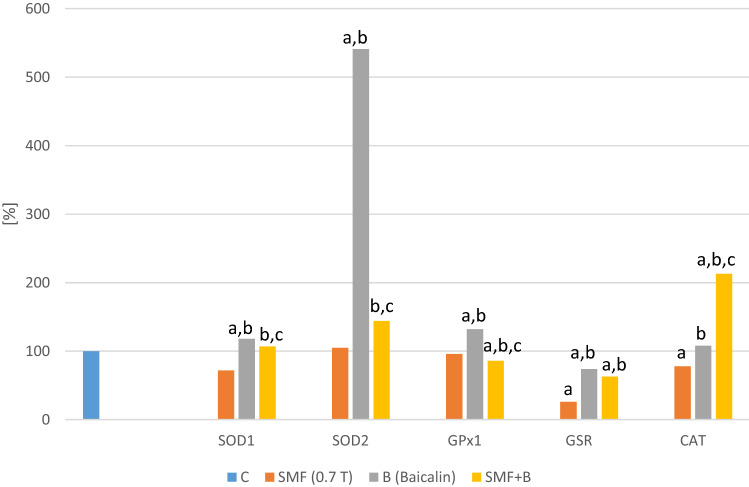
Effect of baicalin and SMF on the antioxidative defense parameters. The results are presented as the mean ± SD; ^a^p < 0.05 vs. C (control); ^b^p < 0.05 vs. SMF; ^c^p < 0.05 vs. B (Baicalin)

**Fig. 2:**
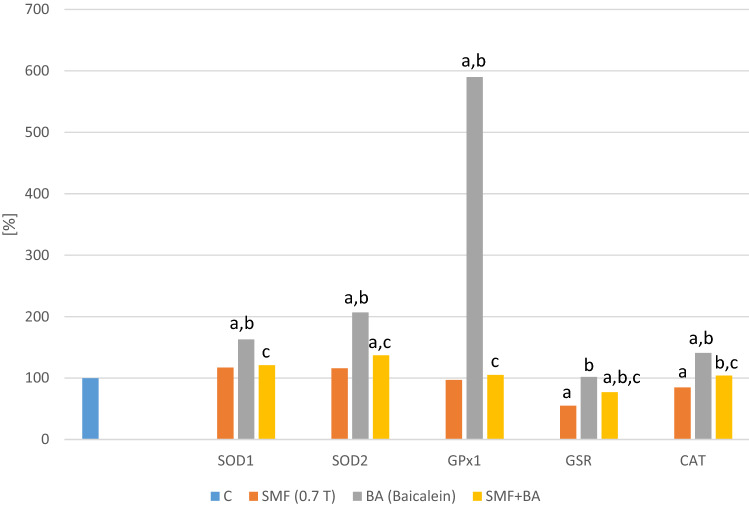
Effect of baicalein and SMF on the antioxidative defense parameters. The results are presented as the mean ± SD; ^a^p < 0.05 vs. C (control); ^b^p < 0.05 vs. SMF; ^c^p < 0.05 vs. BA (Baicalein)

There was no significant difference in the expression of the *SOD1*, *SOD2* or *GPX1* genes in the melanoma cell cultures that had only been exposed to a static magnetic field (0.7 T) (Figs. 1, 2). The addition of the studied flavones to the cell cultures at a concentration of 50 µmol/L resulted in a statistically significant increase (p = 0.0012; p = 0.02; p = 0.003—baicalin; p = 0.022; p = 0.007; p = 0.003—baicalein) in the expression of the *SOD1*, *SOD2* and *GPX1* genes compared to the control cell cultures. On the other hand, the simultaneous exposure of the melanoma cells to an SMF and baicalin or baicalein reduced their mRNA levels (p = 0.007; p = 0.009; p = 0.004—SMF + Baicalin; p = 0.0048; p = 0.01; p = 0.033—SMF + Baicalein) compared to the cultures to which only baicalin or baicalein had been added. The co-exposure of the cells to an SMF and flavones normalized the mRNA levels of the studied genes compared to the control cultures.

### Effect of baicalin or baicalein and an SMF on the activity of the antioxidant enzymes

**Fig. 3:**
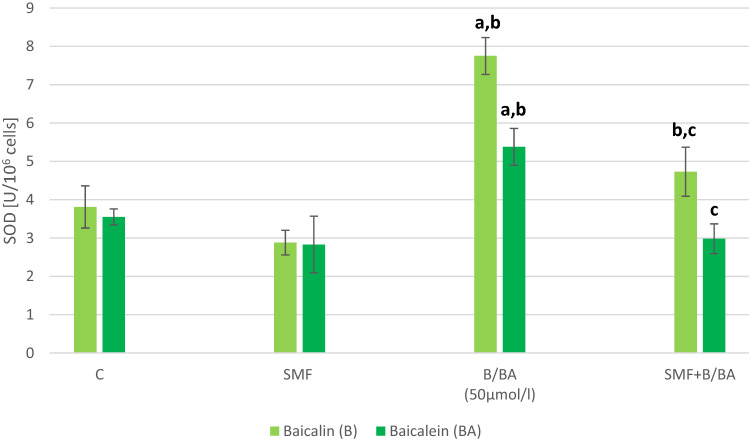
Effect of baicalin or baicalein and SMF on SOD activity in melanoma cell cultures. The results are presented as the mean ± SD; ^a^p < 0.05 vs. C (control); ^b^p < 0.05 vs. SMF; ^c^p < 0.05 vs. BA (Baicalein)/B(Baicalin)

**Fig. 4:**
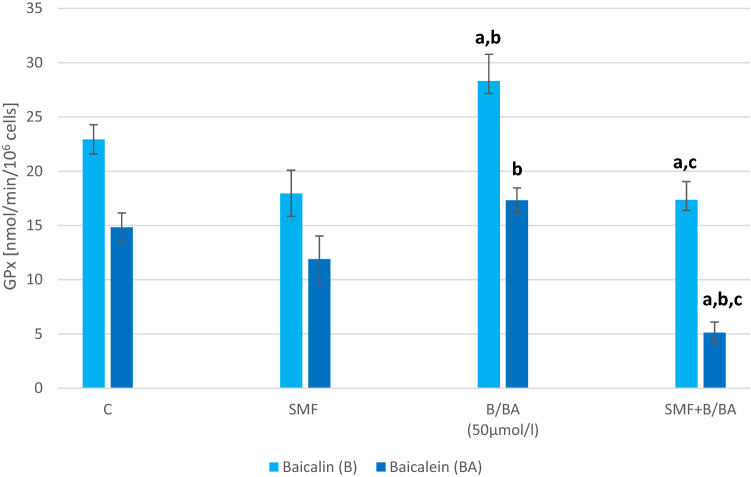
Effect of baicalin or baicalein and SMF on GPx activity in melanoma cell cultures. The results are presented as the mean ± SD; ^a^p < 0.05 vs. C (control); ^b^p < 0.05 vs. SMF; ^c^p < 0.05 vs. BA (Baicalein)/B(Baicalin)

**Fig. 5:**
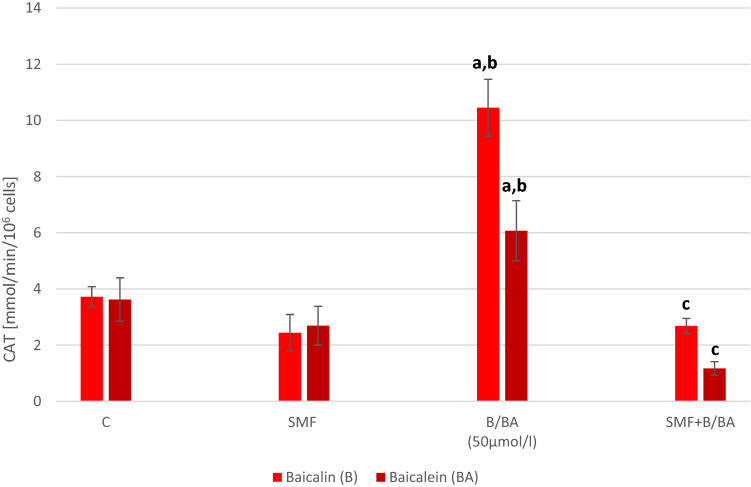
Effect of baicalin or baicalein and SMF on CAT activity in melanoma cell cultures. The results are presented as the mean ± SD; ^a^p < 0.05 vs. C (control); ^b^p < 0.05 vs. SMF; ^c^p < 0.05 vs. BA (Baicalein)/B(Baicalin)

There was no statistically significant difference in the SOD, GPx and CAT activity in the melanoma cell that had only been exposed to an SMF (Figs. 3, 4, 5). In the cultures to which baicalein or baicalin had been added, there was a statistically significant increase in the activity of the antioxidant enzymes (p = 0.002; p = 0.0015; p = 0.004—baicalin; p = 0.002; p = 0.004; p = 0.007—baicalein). The cultures with the addition of baicalin showed an increase in the activity of SOD by about 80%, GPx by about 20% and CAT by about 115% compared to the cultures without the addition of baicalin. The cultures with the addition of baicalein showed an increase in the activity of SOD by about 44% and CAT by about 52% compared to the cultures without the addition of baicalein. The GPx activity decrease about 15% in cultures with baicalein. The simultaneous exposure of the melanoma cells to an SMF and the studied flavones caused a statistically significant decrease in the SOD, GPx and CAT activity compared to the control cultures (p = 0.012; p = 0.017; p = 0.019—SMF + Baicalin; p = 0.022; p = 0.019; p = 0.017—SMF + Baicalein).

## Discussion

In recent years, there has been an increase in the consumption of drugs and dietary supplements. This phenomenon is noticeable both among sick and healthy people. A balanced diet that provides the necessary nutrients in the amounts that are in accordance with the applicable standards should not be replaced by the consumption of one pill that is a panacea for all deficiencies. Antioxidants are a group of substances that are found in food, mainly of foods of a plant origin, and people who eat foods that are rich in antioxidants are healthier. Positive research results and TV commercials have increased the popularity of dietary supplements that contain antioxidants. Currently, more than half of the adults in high-income countries are taking these supplements in the hope of improving their health and preventing aging. Unfortunately, research results show that consuming dietary supplements that are rich in antioxidants does not translate into the prevention of cancer or cardiovascular diseases, and it also does not extend life. Moreover, combining various antioxidants such as β-carotene, vitamins A and E increases the risk of death when the average daily requirement for these substances is exceeded. Moreover, it is not known whether the large amounts of antioxidants that are supplied to the body via dietary supplements will have pro-oxidative and cytotoxic effects on cells [14, 15].

Baicalein and its aglycone baicalin are commonly found in dietary supplements and cosmetic preparations often in doses that could cause a cytotoxic effect. In this study, we used the studied flavones at a concentration that were proven to be cytotoxic to melanoma cells, but not cytotoxic to skin fibroblasts in our previous study [10]. In the studies of Chan et al. [16], the cytotoxic effect of baicalin at concentrations of 150 µmol/L and higher was also observed on prostate cancer cell cultures. The studies by Zhu et al. [17] showed that the cytotoxic effect of baicalin on human chondrosarcoma cells was dependent on both the concentration of the tested flavone and the exposure time. For the cultures that were conducted for 24 h, baicalin at a concentration of 54 µmol/L caused a cytotoxic effect. Additionally, the studies by Yang et al. [18] demonstrated the cytotoxic effect of baicalin on the neoplastic cells of primary liver cancer. This antitumor effect was observed at concentrations of 40 µmol/L and higher.

The results that were obtained in this study indicate that the incubation of melanoma cells to which baicalin and baicalein had been added was accompanied by an increase in the activity of SOD, GPx and CAT. It should be emphasized that these changes were significantly dependent on the chemical form of the added flavones. The observed increase in the SOD activity was probably caused by an increased concentration of the substrate superoxide anion. At the same time, an increase in the activity of catalase was observed as catalase is involved in the reaction of hydrogen peroxide decomposition when its concentration in a cell is high. While a few reports have suggested that if the number of free radicals is small, there is an increase in the activity of the antioxidant enzymes, in particular SOD and GPx, while a high level of ROS leads to the failure of the enzymatic defense mechanisms, which results in a decrease in the activity of the antioxidant enzymes [19, 20]. In the present study, it was found that the addition of the studied flavones caused oxidative stress in the melanoma cells, for which there was also an increase of the *SOD1*, *SOD2*, *GPX1* and *CAT* gene expression. Although the anticancer activity of baicalin may involve a pro-oxidant mechanism, these compounds also cause a depletion of the GSH content in human hepatoma cells [21]. Kong et al. [22] investigated the effect of baicalin on the bladder cancer cells 5637 and KU-19-19 and found that baicalin performed its anticancer activity by inducing apoptosis and cell death in bladder cancer cells. Baicalin-induced ferroptotic cell death in vitro and in vivo, which is accompanied by an accumulation of reactive oxygen species (ROS) and the enrichment of intracellular chelate iron. They also detected the expression of several iron regulatory proteins. Western blotting showed that increased transferrin, phosphorylated histone H2AX (γ-H2AX), P53, the tumor suppressor P53 binding protein 1 (53BP1) and decreased FTH1 were found in baicalin-treated bladder cancer cells. Moreover, many studies have shown that baicalin induces the apoptosis of cancer cells by increasing the expression of the pro-apoptotic protein Bax [23–25].

Results of in vitro studies have also shown that the sensitivity of cells to an SMF depends on many factors such as the SMF induction, the duration of enrichment exposure to it and the type of cells that are used for the studies. It is also difficult to compare test results due to the use of different sources to emit a static magnetic field. This field can be generated by either magnetic coils or magnetic neodymium disks that are placed in Petri dishes or by permanent magnets. In our work, in the melanoma cells that had only been exposed to a static magnetic field with an induction of 0.7 T, there was no disturbance in their oxidation–reduction balance. This was evidenced by the lack of changes in the activity of the antioxidant enzymes as well as the mRNA levels. In the studies of Vergallo et al. [26], human neuroblastomas were treated with a 0.2 T SMF and after two hours of treatment, the cell viability decreased 30% due to the overexpression of the caspase-3 protein. In another study, Li et al. [27] treated human hepatoma cell lines with an SMF with an induction 0.2 T for 3 and 6 days. After 6 days, apoptosis was induced in the BEL-7402 cells, but these treatment conditions had no influence on HepG2 cells.

The main aim of our research was to evaluate the interaction of the studied flavones and an SMF on the oxidative-reduction homeostasis of melanoma cells. In the present study, it was found that the melanoma cell cultures that had simultaneously been exposed to the baicalin or baicalein and an SMF normalized the activity of the antioxidant enzymes and the mRNA level compared to the cultures that had only been treated with flavonoids. As a result of the interaction between an SMF and the flavones, the anticancer effect baicalin and baicalein decreased. The antioxidant properties of flavonoids involve the presence of 2,3-unsaturation in conjugation with a 4-oxo group in the C-ring, the amount of the hydroxyl groups in the B-ring and the 5-hydroxy group in the A-ring. Baicalin and baicalein have the 2,3-unsaturation and the 4-oxo in the C-ring and hydroxyl group in the A-ring [28, 29]. Studies by Kotani et al. [30] and Torbet and Ronziere [31] indicated that exposure to a strong static magnetic field of 1 T can result in a change in the orientation of the macromolecules such as, e.g., collagen but Panczyk et al. [32] suggested that it is very unlikely that a static magnetic field could lead to any changes in the structure of small molecules because Lorentz forces do not affect ions or larger molecules in solutions. Recent studies showed that SMF have a strong influence on the reactivity of chemotherapeutic drugs, which can decrease drug dosage and its side effects [33]. The synergistic effects of an 8.8 mT SMF and cisplatin to chronic myelogenous leukemia cells was observed in the studies of Chen et al. [34] and the studies of Babincova et al. [35], cisplatin, an SMF and radiation induced hyperthermia on the lung carcinoma cell line. In our study, the antagonistic effect of an SMF and flavones was observed. The observed differences with the studies of other authors may result from various sources and various flux densities of static magnetic field. Difficulty in comparing test results also results from the use of different sources emitting a static magnetic field. This field can be generated both by magnetic coils or magnetic neodymium disks placed in Petri dishes, and by permanent magnets. In our study were used special magnetic test chambers with SMF, in which cell culture flasks were placed. In turn, in other report the compact electromagnets were applied.

To summarize, we showed that baicalin and baicalein have probably anti-cancer properties as they disrupt redox homeostasis in melanoma cells as well as increase gene expression of the antioxidant system. Further research is needed to confirm the anti-cancer properties of baicalin and baicalein. There was also an antagonistic interaction between the studied flavones and a static magnetic field, which caused a decrease in the anticancer effects of baicalin and baicalein.
